# A Rare Case of Hypereosinophilic Syndrome-Induced Shower Thrombus Responsive to Nilotinib

**DOI:** 10.7759/cureus.8341

**Published:** 2020-05-28

**Authors:** Elise Lambird, Dharti Patel, Arun Amble, Elizabeth Henderson, Salman Muddassir

**Affiliations:** 1 Internal Medicine, Oak Hill Hospital, Brooksville, USA; 2 Internal Medicine, Hospital Corporation of America West Florida GME Consortium, Brooksville, USA

**Keywords:** hyper-eosinophilia syndrome, nilotinib, cardio vascular disease

## Abstract

Hypereosinophilic syndrome (HES) is a rare clinical disease that affects 0.036/100,000 patients, with a minority of patients having associated genetic markers which can encompass PDGFRA/B or FGFR1 mutations. The prognosis is dependent on the timing of diagnosis and early treatment, with a mortality rate ranging from 48% to 75% if there is a delayed diagnosis. Eosinophilic myocarditis is characterized by invasion of the myocardium with eosinophils. Myeloid neoplasms are a rare, but known cause of HES induced myocarditis. Signs and symptoms can range from being asymptomatic to retrosternal pain, arrhythmias, and even sudden death. HES myocarditis is a diagnosis of exclusion that is made via endomyocardial biopsy. Peripheral eosinophilia is the only specific sign to suggest eosinophilic myocarditis with traditional biomarkers, electrocardiogram, and echocardiogram. Treatment modalities include systemic corticosteroids and symptomatic management. Complications from HES myocarditis may include embolic events, eosinophilic vegetations, and dysrhythmias, or conduction disturbances. We present a case of a 62-year-old male who initially presented with epigastric pain, and then suffered a myocardial infarction. After testing, the probable diagnosis of eosinophilic myocarditis was made. His clinical course was complicated by the development of shower thrombus associated with acute encephalopathy. Although HES has classically been treated with imatinib, in this case, an alternative biologic agent was used, resulting in a good prognosis and ultimate patient survival. This case details the importance of early clinical suspicion, diagnosing the condition, and early initiation of treatment to prevent worsening clinical status.

## Introduction

Hypereosinophilic syndrome (HES) is a rare disorder with variable and unpredictable clinical presentation. Hypereosinophilia is defined as a condition wherein the eosinophils are >1.5 x 10^9/L on two separate occasions, or a single elevated reading with associated bone marrow eosinophil cellularity >20% [1]. Common presenting complaints include fatigue, cough, angioedema, fever, and rhinitis. One complication that can arise from HES is eosinophilic myocarditis (EM), which is characterized by invasion of the myocardium with eosinophils. While most occurrences of EM are idiopathic, rare cases have been associated with myeloid neoplasm. In the case of primary eosinophilia, it is prudent to consider genetic causes of myeloid neoplasm, including abnormalities of platelet-derived growth factor receptor α (PDGFRA), platelet-derived growth factor receptor β (PDGFRB), fibroblast growth factor receptor 1 (FGFR1), or PCM1-JAK2 [2]. 

HES is defined as hypereosinophilia in the presence of end organ damage, regardless of whether hypereosinophilia is primary or secondary [3]. Prior to the advent of tyrosine kinase inhibitors, HES-related endomyocardial fibrosis and thromboembolic events resulted in extremely high mortality rates [4]. Fortunately, patients with PDGFRA mutations have responded rapidly and often achieved complete hematologic remission with imatinib [5]. Although imatinib remains the gold standard, more recent literature suggests that nilotinib, sorafenib, dasatinib, and midostaurin are possible alternatives [6]. If the response to these medications is not adequate, corticosteroids can be added to further suppress the immune response, as was the case with this patient.

## Case presentation

A 62-year-old Caucasian male with a past medical history of coronary artery disease, five stents, hypertension, and hyperlipidemia presented to the emergency department for abdominal pain. Approximately two weeks prior to this presentation, bone marrow biopsy was obtained due to persistent leukocytosis as high as 33.8 (range 4.5-11.0) with 35% (range 0%-7%) eosinophils. Imaging during the previous admission demonstrated splenomegaly but was otherwise unremarkable. The patient was directed to follow up in an outpatient facility for results and further management. Unfortunately, he presented to the emergency department with a chief complaint of abdominal pain before admission to the outpatient facility. 

On initial exam, the patient was chronically ill and had mild epigastric tenderness to palpation. Additionally, he had many small wounds approximately 3 mm in size with surrounding excoriations. The patient attributed the wounds to ‘bugs in his house’, though he also noted that he had never seen any bugs anywhere. Although the patient was alert and oriented to person, place, time, he sometimes answered questions in an eccentric manner. Family at the bedside indicated that this behavior was baseline for the patient. 

**Figure 1:**
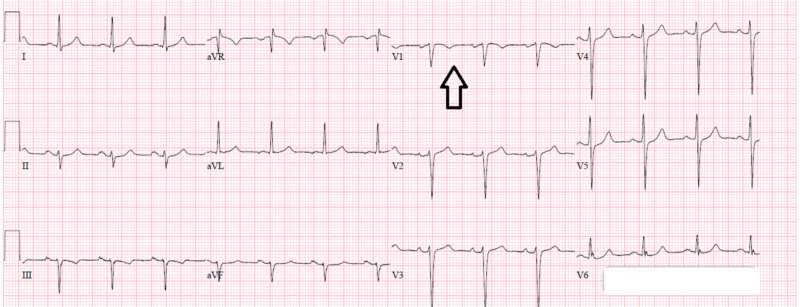
EKG showing non-ST-elevation myocardial infarction (NSTEMI), indicated by inverted T waves (see arrow)

**Figure 2:**
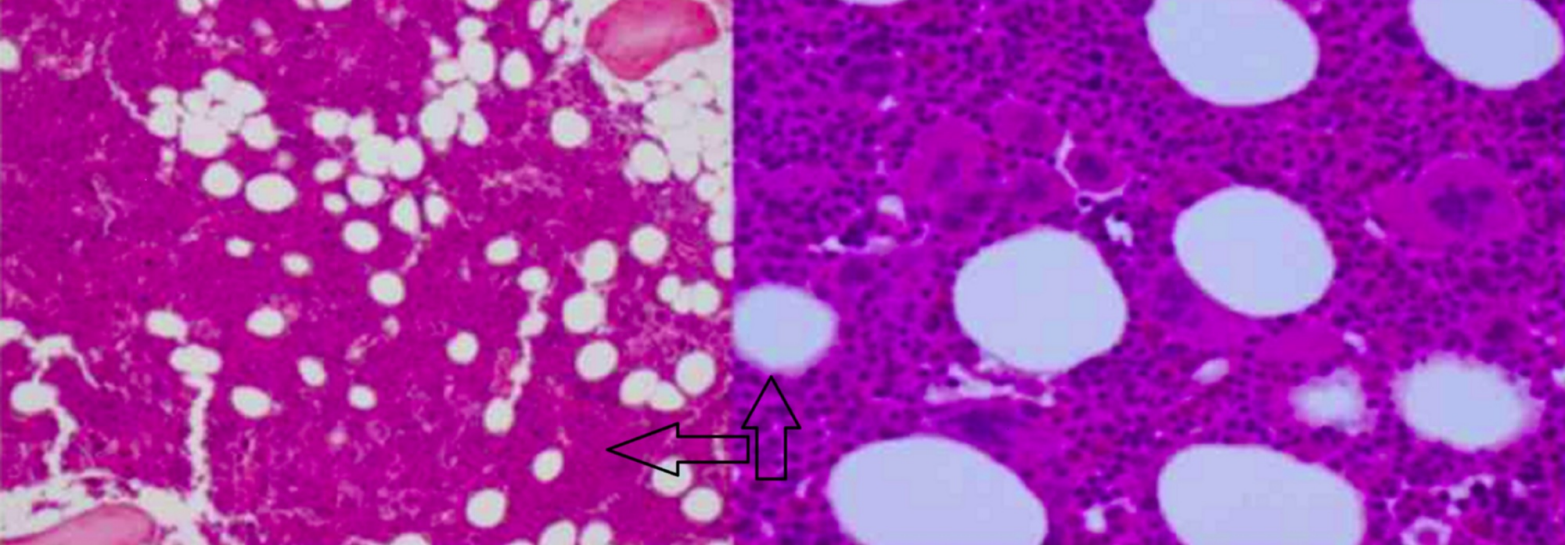
Bone marrow biopsy, negative for BCR-ABL1 and JAK 2 mutation (see arrow)

**Figure 3:**
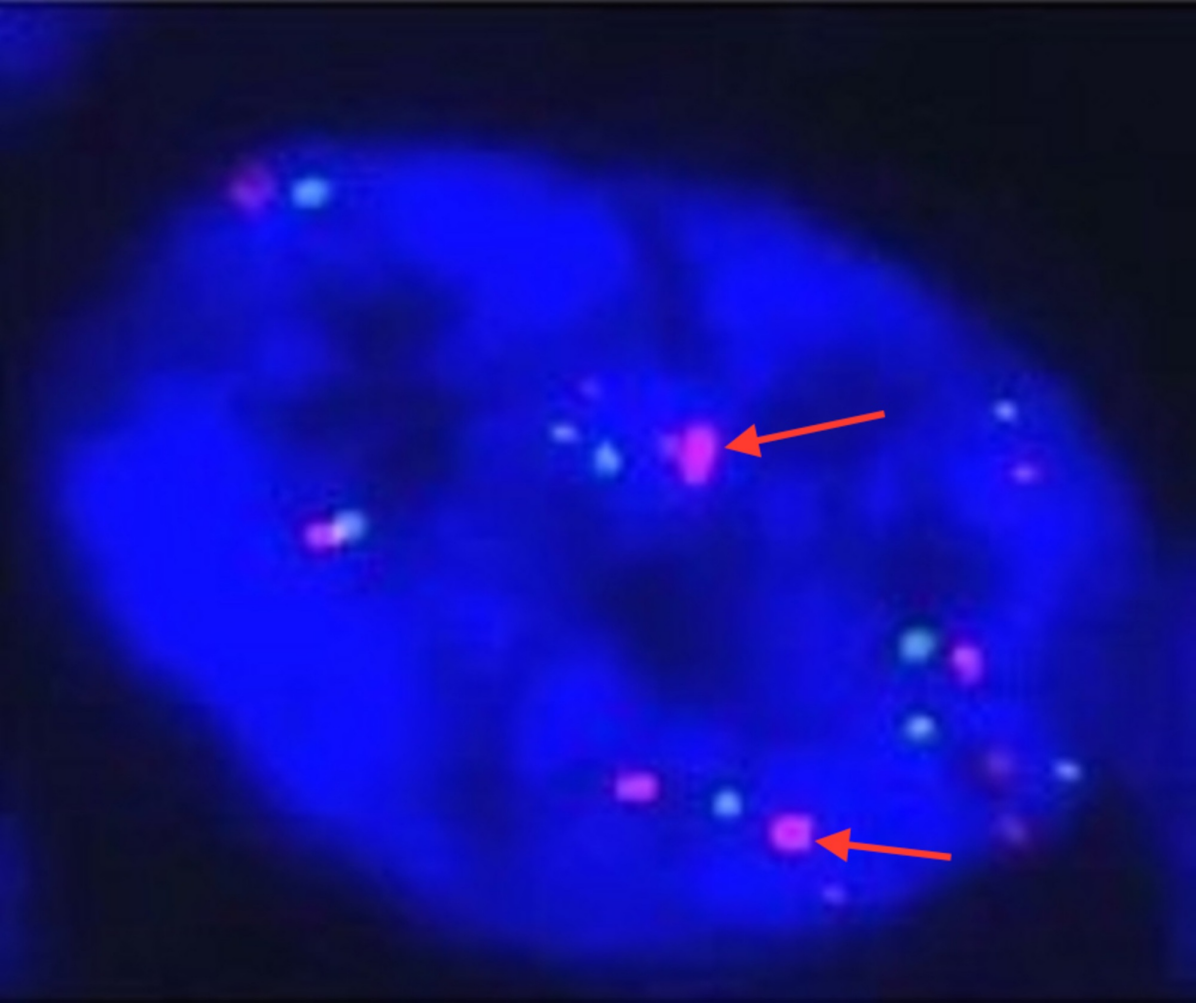
Fluorescence in situ hybridization (FISH) analysis The arrows in the image indicate the presence of platelet-derived growth factor receptor A (PDGRFA) mutation.

At the time of admission, troponins were found to be 1.45 (range 0.00-0.050) without ischemic changes on EKG. The patient was diagnosed with non-ST-elevation myocardial infarction (NSTEMI) and was started on a heparin drip, nitroglycerin drip, aspirin, and atorvastatin (Figure 1). Leukocytosis persisted with WBC 32.8 (range 4.5-11.0) and 56% (range 0-7) eosinophils were present on initial workup. The patient additionally had mild thrombocytopenia with a value of 100 (range 125-400). Peripheral smear demonstrated marked leukocytosis with peripheral eosinophilia and immature granulocytes consistent with a leukemoid reaction. Pathology noted a diminished quantity of platelets. The bone marrow biopsy from the patient’s previous admission was evaluated for JAK2 mutation and BCR-ABL1; additionally, FISH analysis was performed for PDGFRA, PDGFRB, FGFR1, and core-binding factor subunit beta (CBFB) (Figures 2-3). The analysis was positive for PDGFRA mutation with 50% cellularity consistent with a myeloid neoplasm. In light of the NSTEMI with concomitant eosinophilia with mild thrombocytopenia, oncology was consulted urgently to both evaluate the performance status of the patient and candidacy for dual antiplatelet therapy. 

On day two of admission, the WBC increased to 32.8 with 76% eosinophils. The troponins continued to trend upwards and eventually peaked at 7.81. It was still unclear whether the troponin production was secondary to eosinophilic myocarditis or type 1 NSTEMI. The patient was started on high-dose methylprednisolone to treat possible myocarditis. Within hours of steroid initiation, there was a sharp decrease in troponin from 7.81 to 4.50. Heparin drip and nitroglycerin drip were discontinued at this time, as the patient was consistently non-compliant and actively refused to be on any drip. Due to progressive thrombocytopenia, heparin-induced thrombocytopenia (HIT) panel was ordered and was subsequently negative. The thrombocytopenia also resolved with discontinuation of the offending medication, pantoprazole. As the patient’s clinical status improved, a delayed diagnostic heart catheterization was planned. 

Unfortunately, on day three of admission, the patient experienced a change in mental status prior to the catheterization. The patient developed mild encephalopathy, which was most apparent when he became disoriented and unable to find his bed when returning from the restroom. MRI of the brain was ordered immediately to rule out stroke. Unfortunately, the patient had extensive shower emboli that were too numerous to count. Neurology was consulted urgently to evaluate the patient. Transesophageal echocardiography (TEE), lipid profile, and ultrasound of the carotids were added to the workup in progress. Clopidogrel was added to the patient’s medication regimen, which already included aspirin and atorvastatin. In the meantime, diagnostic catheterization was performed, and the patient was diagnosed with 80% occlusion of the LAD. At this junction, the patient was started on nilotinib to manage the ongoing hypereosinophilia on the basis of several case studies that demonstrated good efficacy [7]. An echocardiogram was performed, and was unremarkable. TEE was performed the following morning and did not support cardiac origin for the emboli. The lipid profile was unremarkable. Ultrasound of the carotid arteries did not demonstrate any significant stenosis in the external carotids. The patient’s encephalopathy resolved over approximately 24 hours. The cardiologist used this window to stent the mid-LAD. Despite these findings, the cardiologist suspected eosinophilic myocarditis and recommended continued treatment for the same. The patient continued with high-dose methylprednisolone followed by a taper. The patient experienced complete remission of eosinophilia over 48 hours following initiation of the nilotinib and steroid combination. Tryptase was elevated at 20.4 (reference range 2.2-13.2) and B12 was elevated >1000 (reference range 211-911) (Table 1). 

**Figure 4:**
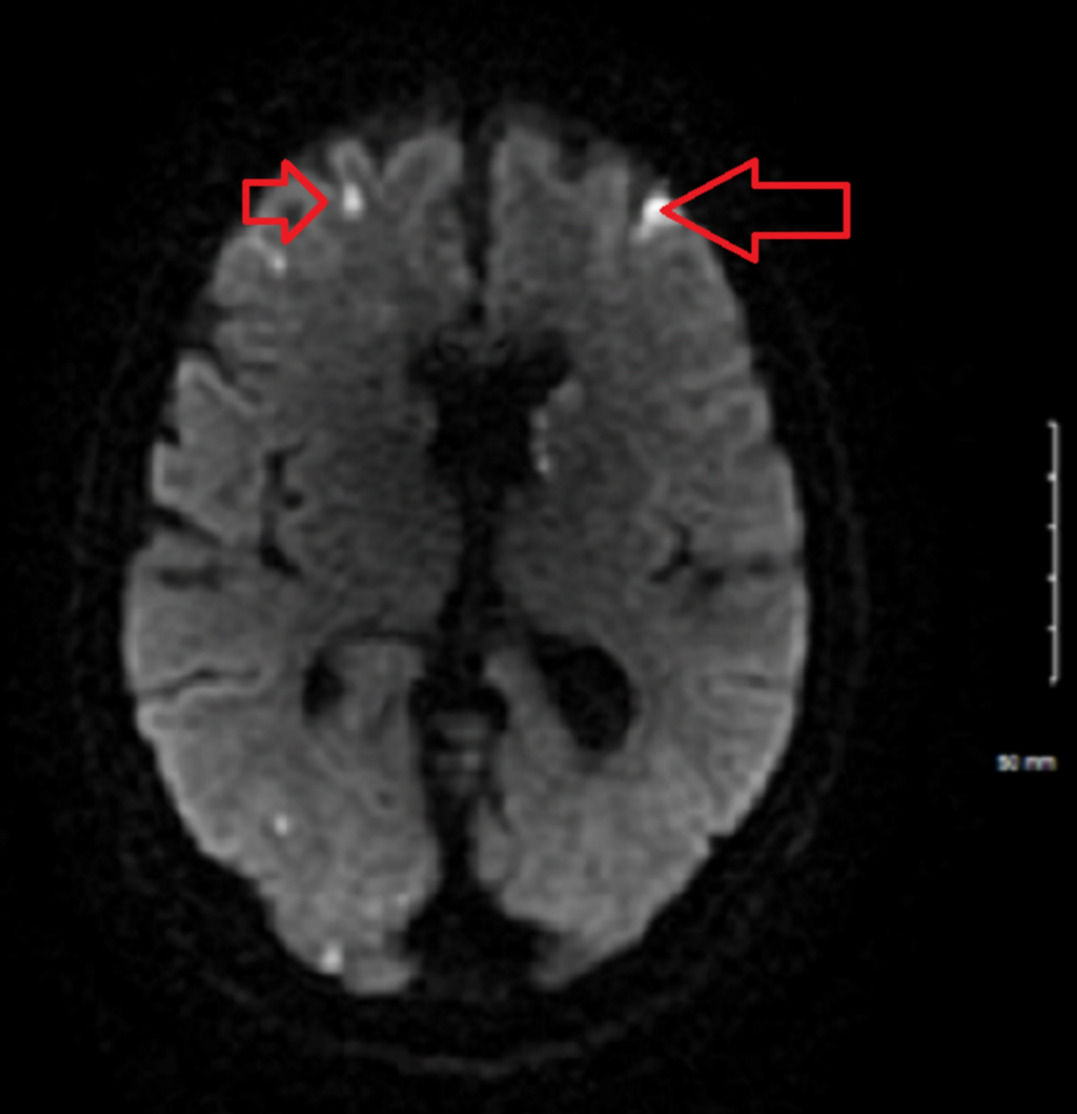
MRI of brain to rule out stroke The arrows indicate the extensive shower emboli found on the MRI.

**Table 1:** Lab values WBC: white blood cells

Labs	Value	Range limits
WBC	32,800	4-11,000
Eosinophils	56%	0-6%
Troponin	1.45	0.00-0.050
Tryptase	20.4	2.2-13.2 ng/ml
B12	>1000	211-911

## Discussion

The normal adult range for eosinophil counts is 20-500 cells/microliter and accounts for 1%-3% of the peripheral blood. Absolute eosinophilia count is used in determining whether eosinophilia is mild (500-1500 cells/microliter), moderate (1500-5000 cells/microliter), or severe (>5000 cells/microliter) [8]. In this case, the absolute eosinophil count of 23,484 with concurrent 50% cellularity on bone marrow biopsy, led to the diagnosis of severe eosinophilia. Recent literature indicates that the incidence and prevalence of hypereosinophilic syndrome is 0.036/100,000 [9]. Genetic aberrance in PDGFRA/B or FGFR1 encompasses 23% of the aforementioned group. In this case, PDGFRA rearrangement was identified as the cause of primary eosinophilia, which led to the diagnosis of chronic eosinophilic leukemia [10]. In the presence of myeloid neoplasm, serum tryptase and B12 levels may be elevated, particularly in the case of PDGFRA or PDGFRB fusion genes [9]. Unfortunately, elevated tryptase levels have traditionally been associated with poor outcomes and increased fibroproliferative damage [11]. However, serum tryptase elevation is also associated with a positive response to imatinib [12].

HES has a heterogeneous clinical presentation with the most common presentations being fatigue, cough, angioedema, fever, and rhinitis [13]. Common hematological findings include anemia and thrombocytopenia, which were present in this case [8]. Eosinophil-mediated myocarditis and subsequent fibrosis is a known complication of HES that occurs with eosinophilic infiltration of cardiac tissue. Eosinophil mediated damage occurs in three distinct phases. In the first stage, degranulation of eosinophils leads to myocardial necrosis. The subsequent stage is associated with hypercoagulability as there is an increase in circulating thrombin. The excess eosinophils are available to bind to thrombomodulin, preventing the formation of the thrombomodulin-thrombin complex. The final phase is heralded by fibrosis developing in tissues [13]. In this case, it is unclear whether the elevated troponins originated from NSTEMI or myocarditis due to both a lack of biopsy and an extensive cardiac history of coronary stenting with previous occlusions. Nonetheless, it was essential to act promptly with regard to steroid administration to prevent further complications.

HES has previously been associated with both thrombotic and embolic strokes [14]. Despite the hypercoagulable state, relatively few cases of shower thrombus have been reported. In early disease, initial small strokes located in the arterial border zones may be precursors to larger strokes in the cortical and subcortical areas [15]. Encephalopathy has long been reported with hypereosinophilia, and is more commonly found in patients with higher eosinophil counts [15].

 It is essential to properly diagnose and risk stratify patients in order to determine the appropriate course of treatment. Poor prognosis is associated with male sex, steroid refractory disease, age greater than 60, hemoglobin <10 g/dl, hepatosplenomegaly, and heart disease [15]. The patient’s splenomegaly, heart disease, age, and male sex were unfortunately not in the patient’s favor. Although patients historically have a three-year survival rate of 12%, this dictum has been altered with new chemotherapeutic agents [15].

## Conclusions

While there are several drugs that can be used in cases like these such as imatinib, nilotinib, sorafenib, dasatinib, and midostaurin, the patient had an excellent response to nilotinib in combination with a corticosteroid that resulted in the complete remission of the eosinophilia. Hypereosinophilic syndrome is a rare condition and it is crucial to be vigilant to prevent further complications affecting various organs. 
